# Efficacy and safety of antibiotic agents in the treatment of rosacea: a systemic network meta-analysis

**DOI:** 10.3389/fphar.2023.1169916

**Published:** 2023-05-11

**Authors:** Wenqin Xiao, Mengting Chen, Ben Wang, Yingxue Huang, Zhixiang Zhao, Zhili Deng, Hongfu Xie, Ji Li, Yan Tang

**Affiliations:** ^1^ Department of Dermatology, Xiangya Hospital, Central South University, Changsha, Hunan, China; ^2^ Hunan Key Laboratory of Aging Biology, Xiangya Hospital, Central South University, Changsha, Hunan, China; ^3^ National Clinical Research Center for Geriatric Disorders, Xiangya Hospital, Central South University, Changsha, Hunan, China

**Keywords:** rosacea, treatment, efficacy, safety, antibiotic, network meta-analysis

## Abstract

**Background:** Antibiotics are considered the backbone of rosacea management, especially for controlling inflammatory papules and pustules. We aim to evaluate the efficacy and safety of varied prescriptions and doses of antibiotics in treating rosacea by network meta-analysis.

**Methods:** In this study, we compared all available randomized controlled trials (RCTs) that have studied systemic and topical antibiotics and placebo in rosacea therapy. We searched databases such as the Cochrane Central Register of Controlled Trials (CENTRAL), MEDLINE, Embase, PubMed, Web of Science, and LILACS for published and unpublished RCTs on ClinicalTrials.gov before April 2023. The primary outcome was the improvement of the Investigator's Global Assessment (IGA) scores, and the secondary outcomes consisted of the improvement of the Patient's Global Assessment (PaGA) scores, Clinician's Erythema Assessment (CEA) scores, and adverse events (AEs). We used Bayesian random effects models for multiple treatment comparisons.

**Results:** We identified 1,703 results through these databases. Thirty-one randomized trials with 8,226 patients were included. The heterogeneity and inconsistency between the trials were low, with a low risk of bias of all trials. Oral doxycycline 40 mg, minocycline 100 mg, and minocycline 40 mg, as well as topical ivermectin and metronidazole 0.75%, were effective in treating papules and pustules, thereby decreasing IGA in rosacea. Among these, minocycline 100 mg ranked top in efficacy. As for improving the PaGA scores, topical ivermectin, metronidazole 1%, and systemic oxytetracycline were effective, of which oxytetracycline worked the best. Both doxycycline 40 mg and metronidazole 0.75% presented no therapeutic effect for erythema. Considering the safety of the agents, systemic application of azithromycin and doxycycline 100 mg significantly increase the risk of AEs.

**Conclusion:** Our review suggests that a high dosage of systemic minocycline is the most effective in treating rosacea phenotypes with papules and pustules with a low risk of AEs. However, there were no sufficient evidence-based data in exploring the influence of antibiotics on erythema. The phenotype of rosacea should be taken into consideration along with benefit and safety when making prescriptions due to AEs.

**Clinical Trial Registration:** NCT(2016): http://cochranelibrary-wiley.com/o/cochrane/clcentral/articles/962/CN-01506962/frame.html NCT(2017): http://cochranelibrary-wiley.com/o/cochrane/clcentral/articles/764/CN-01565764/frame.html

## 1 Introduction

Rosacea is a chronic inflammatory skin disease that primarily affects the central face, especially the chin, cheeks, and nose (van Zuuren, 2017). It is commonly seen among women aged 20–50 years and involves nearly 5.46% of people worldwide (Gether et al., 2018). The featured manifestations include inflammatory papules/pustules, fixed central facial erythema, telangiectasia, phymatous changes, and abnormal sensations such as burning, stinging, and dryness. Social interaction and psychological health should be concerned due to the cosmetic changes among rosacea populations (Deng et al., 2018; Tan et al., 2018).

The management of rosacea has long been considered tricky due to its characteristics of repeated attacks and irritability. Hence, a safe and efficient strategy is greatly essential for long-term application. According to the recommendation from the National Rosacea Society Expert Committee and global ROSacea COnsensus (ROSCO) panel in 2017, a wide range of topical and systemic antibiotics was considered the first-line treatment of rosacea. Among these, a variety of topical and systemic antibiotics were approved by the United States Food and Drug Administration targeting inflammatory papules/pustules of rosacea, which included ivermectin, metronidazole, doxycycline, and minocycline of different doses (Schaller et al., 2017; van Zuuren, 2017; Thiboutot et al., 2020). Interestingly, some of them could relieve flushing or erythema. Hence, we aimed at ranking the antibiotics in treating both papules and pustules and flushing/erythema.

The side effects of the therapy are worth deliberative consideration, apart from the curative effect when making the prescription. Topical antibiotics of different agents may cause diverse degrees of skin irritation, pruritus, stinging, or even blisters and ulceration. Moreover, systemic antibiotics might exert more adverse effects. For instance, oral doxycycline and tetracycline may result in side effects such as gastrointestinal discomfort, photosensitivity, morbilliform exanthem, and other discomforts (Smilack, 1999; Jacob and Cohen, 2020). It is important to balance the treatment efficacy and side effects when making the decision among the varieties of antibiotics based on the phenotype.

To date, numerous randomized controlled trials (RCTs) have been conducted worldwide on comparing the efficacy and safety of antibiotics in treating rosacea. However, few research works have concentrated on ranking the antibiotics based on all the evidence. There still lacks solid evidence to make the decision, since the scattered RCTs conducted on diverse study populations with different designs have to be unified to reduce the inconsistent results. Besides, most of these trials compare antibiotics with placebo, lacking the head-to-head comparison between different interventions. In addition, traditional systemic reviews or meta-analyses are restricted to traditional pairwise comparisons, considering only direct evidence, and fail to measure the effectiveness and safety between more types or dosages of antibiotics in treating rosacea, while combining all potential direct and indirect evidence. Hence, there are numerous limitations in clinical practice due to the abovementioned reasons.

To provide comprehensive information, we conducted this network meta-analysis by integrating all the available direct and indirect evidence to compare the effectiveness and safety of various prescriptions and dosages of antibiotics administrated in rosacea.

## 2 Methods

### 2.1 Selection criteria

We included RCTs from seven databases to compare the efficiency of any of the following interventions in rosacea: topical and systemic antibiotics and placebo. We excluded studies published as abstracts only or with no outcome of interest. The patients enrolled were all >18 years of age without any other disease and had stopped additional medication by the time that the trial started.

### 2.2 Search strategy

Up to April 2023, we searched the Cochrane Central Register of Controlled Trials (CENTRAL), MEDLINE, Embase, PubMed, Web of Science, and LILACS for published RCTs, as well as unpublished RCTs with results available on ClinicalTrials.gov, using the search terms: rosacea and (antibiotic* or antibacterial or anti-infect* or amoxicillin or amphotericin b or ampicillin or calcimycin or cephalosporin* or cephalothin or cephamycin* or chloramphenicol or dactinomycin or doxycycline or erythromycin or fluoroquinolone* or gentamicin* or kanamycin or minocycline or neomycin or oxytetracycline or penicillin or streptomycin or tetracycline or vancomycin or macrolide* or quinolone* or trimethoprim or augmentin or cotrimoxazole or clavulin* or ceftin* or ivermectin*).

### 2.3 Selection of trials and extraction of data

Two reviewers individually selected the trials depending on the selection criteria and extracted the information. Disagreements were solved by discussion. By reading the full text, we extracted the study design, sample size, details of intervention (such as dosage, forms, and duration of treatment), baseline characteristics, outcomes at the endpoint, and risk of bias with criteria.

### 2.4 Outcomes

The primary outcome was the proportion of patients with the Investigator's Global Assessment (IGA) scored at 0 or 1 in a 5-point scale at the end of treatment.

The secondary outcomes included 1) the proportion of patients with the Patient's Global Assessment (PaGA) scores valued with at least a one-point improvement; 2) the mean changes of the Clinician' Erythema Assessment (CEA) scores from the baseline to end; and 3) the number of patients who had acquired adverse events (AEs) during the trials. We extracted the data at the baseline and end of treatment.

### 2.5 Risk of bias

The methodological quality of trials included was measured according to the “Risk of Bias” in the Cochrane handbook. The assessment was completed independently by two authors.

### 2.6 Statistical analysis

Network meta-analysis was conducted in the R version 3.6 (R Foundation; packages meta, gemtc, coda, and rjags) using the Just Another Gibbs Sampler (JAGS) version 4.3.0. We used Bayesian random effects models for multiple treatment comparisons while preserving the direct randomized comparisons within each trial. We have presented results as the mean difference (MD) or odds ratio (OR) with 95% confidence intervals using the forest plot, with horizontal lines representing MD or OR with 95% CI of interventions in each trail and circles representing the combination results of each intervention. The rank plot was for displaying the rank probabilities of interventions (probability of being the best or worst) in each endpoint according to the estimated effect size. We set a 5% level of statistical significance (*p*-value).

We calculated the heterogeneity of the intervention effect by measuring I^2^. I^2^ >50% represents substantial heterogeneity. We also measured the incoherence between direct and indirect comparisons by using the node-splitting approach contrasting estimates from both direct and indirect evidence.

## 3 Results

### 3.1 Characteristics of included studies

**FIGURE 1:**
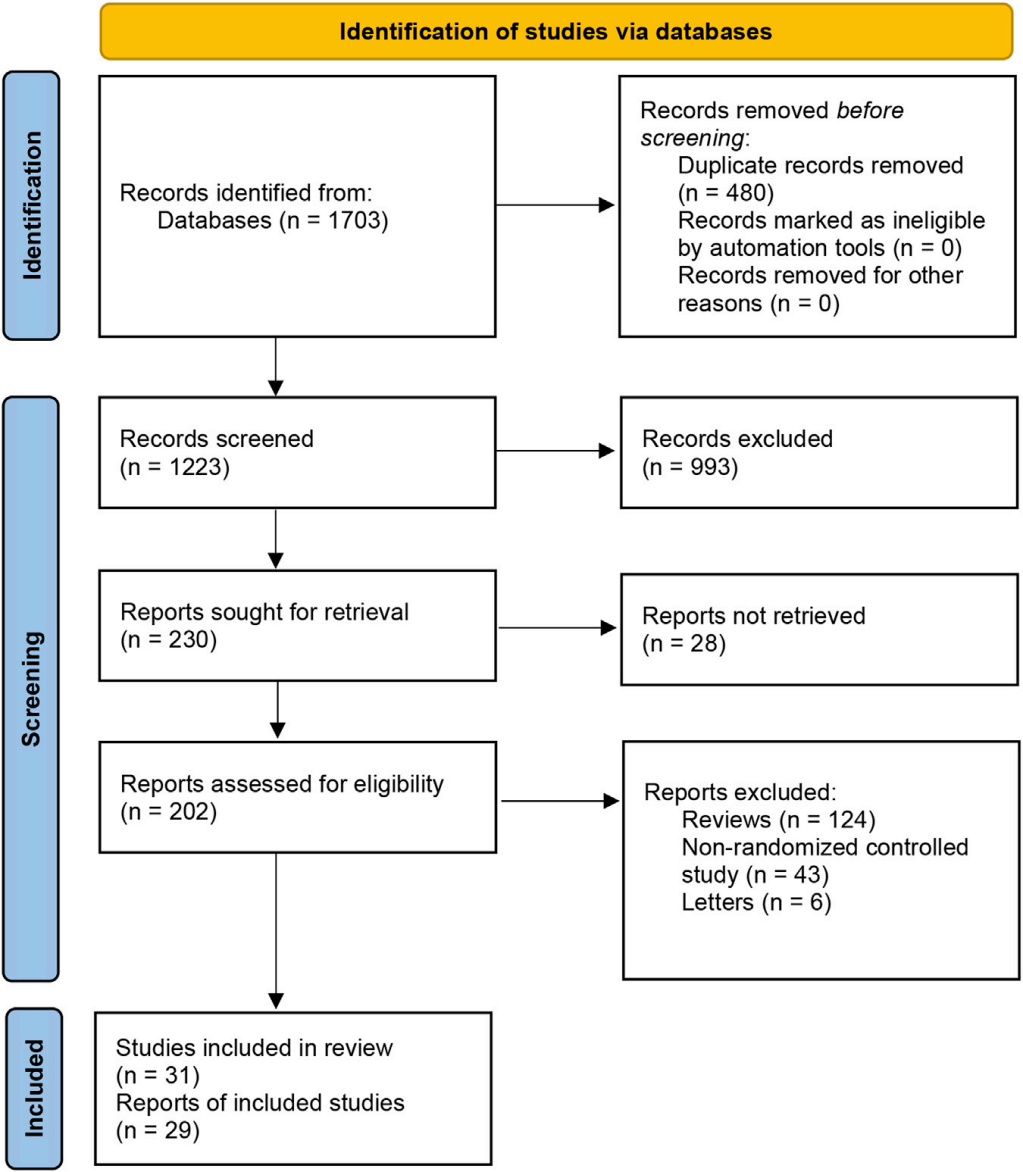
Summary of study retrieval and identification. Preferred Reporting Items for Systematic Reviews and Meta-Analyses (PRISMA) flow diagram of the literature search.

We searched for all the trials and systemic reviews concerning the treatment of rosacea on the Cochrane Central Register of Controlled Trials (CENTRAL), Medline, Embase, PubMed, Web of Science and LILACS according to the search strategy we have mentioned above. We identified 1,703 records through these databases in total and excluded 480 duplicates and 993 records after carefully reading each title and abstract. We then excluded 28 reports for which we did not find full texts. After browsing the full text of the remaining articles based on our inclusion/exclusion criteria, w excluded 124 reviews, 43 non-randomized controlled studies, and 6 letters. Finally, we took into account 29 reports, which included 31 randomized control studies (Figure 1).

These 31 RCTs included 8,226 patients in total, prescribing with different varieties and dosages of antibiotics that included systemic treatments (ampicillin, azithromycin, doxycycline 100 mg, doxycycline 40 mg, minocycline 100 mg, minocycline 40 mg, minocycline 20 mg, oxytetracycline, and tetracycline), topical treatments (clindamycin 1% bid, clindamycin 1% qd, clindamycin 3%, erythromycin, ivermectin, metronidazole 1%, metronidazole 0.75%, minocycline 3%, minocycline 1.5%, and minocycline 1%), and placebo. The direct comparison of 20 interventions demonstrated in 31 research studies has been presented as network plots. Each node of the circle represents one kind of form and dosage of antibiotic, and each direct comparison is represented as the line connecting two nodes. The size of the nodes is proportional to the number of randomly assigned participants. The width of the lines is proportional to the number of randomized clinical trials directly comparing the two treatments. We estimated the primary outcomes of the proportion of patients with the IGA score of 0 or 1 from the baseline to end, secondary outcomes that included the proportion of patients with improved PaGA, and mean changes of CEA to assess the effectiveness of antibiotics in treating rosacea. We also measured the number of patients with AEs to evaluate the safety of the antibiotics. The proportion of women was between 43.55 and 82.3%. The follow-up duration ranged from 6 to 40 weeks, and the mean age of the patients involved ranged from 37 to 59.78 years. The main study and characteristics of the included trials are described in Table 1.

**TABLE 1:** Characteristics of included studies.

Study	Interventions	Number of patients	Number of women <%>	Follow-up	Mean age (years)
Akhyani et al. (2008)	Azithromycin *vs.* doxycycline <100 mg>	67	30 <44.78>	3 months	47.93
Bitar et al. (1990)	Placebo *vs.* metronidazole <1%>	100	59 <59>	8 weeks	50.6
Bjerke et al. (1989)	Placebo *vs.* metronidazole <1%>	97	53 <54.6>	60 days	47
Cai et al. (2002)	Placebo *vs.* metronidazole <0.75%>	62	27 <43.55>	12 weeks	37
Dahl et al. (2001)	Metronidazole <0.75> *vs.* metronidazole <1%>	72	51 <70.8>	12 weeks	46
Fowler et al. (2007)	Placebo *vs.* doxycycline <40 mg>	72	56 <77.78>	16 weeks	47
Gold et al. (2020) a	Placebo *vs.* minocycline <1.5%>	751	541 <72.0>	12 weeks	49.2
Gold et al. (2020) b	Placebo *vs.* minocycline <1.5%>	770	533 <69.22>	12 weeks	50.9
Huang et al. (2014)	Placebo *vs.* doxycycline <40 mg>	170	121 <71.2>	12 weeks	49.1
Koçak et al. (2002)	Placebo *vs.* metronidazole <0.75%>	63	48 <80>	60 days	51
Linden et al. (2017)	Doxycycline <40 mg> *vs.* minocycline <100 mg>	80	59 <74>	16 weeks	46
Marks et al. (1971)	Placebo *vs.* ampicillin *vs.* tetracycline	56	29 <51.8>	6 weeks	47.8
Martel et al. (2017) a	Placebo *vs.* clindamycin <0.3% Qd> vs. clindamycin <1% Bid> vs. clindamycin <1% Qd>	416	311 <74.75>	12 weeks	47.9
Martel et al. (2017) b	Placebo *vs.* clindamycin <1% Bid>	213	147 <69.01>	12 weeks	48.12
Miyachi et al. (2021)	Placebo *vs.* metronidazole <0.75%>	130	107 <82.3>	12 weeks	47.8
Monk et al. (1991)	Metronidazole <0.75%> *vs.* oxytetracycline	33	16 <48.48>	9 weeks	49
Mrowietz et al. (2018)	Placebo vs. minocycline <1.5%> *vs.* minocycline <3%>	232	145 <62.5>	12 weeks	52.2
NCT02840461 (2016)	Placebo *vs.* ivermectin	630	449 71.3>	12 weeks	51.1
NCT (2017)	Placebo *vs.* doxycycline <40 mg> *vs.* minocycline <20 mg> *vs.* minocycline <40 mg>	205	124 <60.5>	16 weeks	50.5
Nielsen (1983) a	Placebo *vs.* metronidazole <1%>	81	49 <60.49>	2 months	47
Nielsen (1983) b	Metronidazole <1%> *vs.* oxytetracycline	51	34 <66.67>	2 months	44
Rosso et al. (2007)	Placebo *vs.* doxycycline <40 mg>	537	375 <69.8>	16 weeks	47.07
Rosso et al. (2007)	Doxycycline <40 mg> *vs.* doxycycline <100 mg>	91	64 <70.33>	16 weeks	44.76
Rosso et al. (2020)	Placebo *vs.* doxycycline <40 mg>	130	86 <66.15>	40 weeks	49.4
Schachter et al. (1991)	Metronidazole <1%> *vs.* tetracycline	125	61 <48.8>	2 months	45.4
Schaller et al. (2020)	Placebo *vs.* doxycycline <40 mg>	273	155 <56.78>	12 weeks	52
Stein et al. (2014)	Placebo *vs.* ivermectin	1,371	925 <67.5>	12 weeks	50.3
Taieb et al. (2015)	Ivermectin *vs.* metronidazole <0.75%>	962	627 <65.2>	16 weeks	51.54
Veien et al. (1986)	Metronidazole <1%> *vs.* tetracycline	76	39 <52>	8 weeks	52.4
Verea et al. (1992)	Erythromycin *vs.* metronidazole <0.75%>	40	27 <67.5>	3 months	59.78
Webster et al. (2020)	Placebo *vs.* minocycline <1%> *vs.* minocycline <3%>	270	189 <70>	12 weeks	51.1

Bid = twice a day; Qd = once a day; a/b represents different studies in one report.

The risk of bias for the included studies was measured by the Cochrane Collaboration tool (Supplementary Figure S1). Most studies were judged to be at a low risk of bias for blinding of participants, personal incomplete outcome data, and selective reporting. Most studies were judged to be at a low or unclear risk of bias for random sequence generation and blinding of the outcome assessment. The risk of bias was determined to be unclear for allocation concealment in most of these trials. The heterogeneity of the primary and secondary outcomes (IGA, PaGA, CEA and AEs) was evaluated, and therewas no statistically significant relative heterogeneity (I^2^ = 51.76024, 0, 0, and 0, respectively) (Supplementary Tables S1–S4).

### 3.2 Primary outcome: Improvement of IGA

**FIGURE 2:**
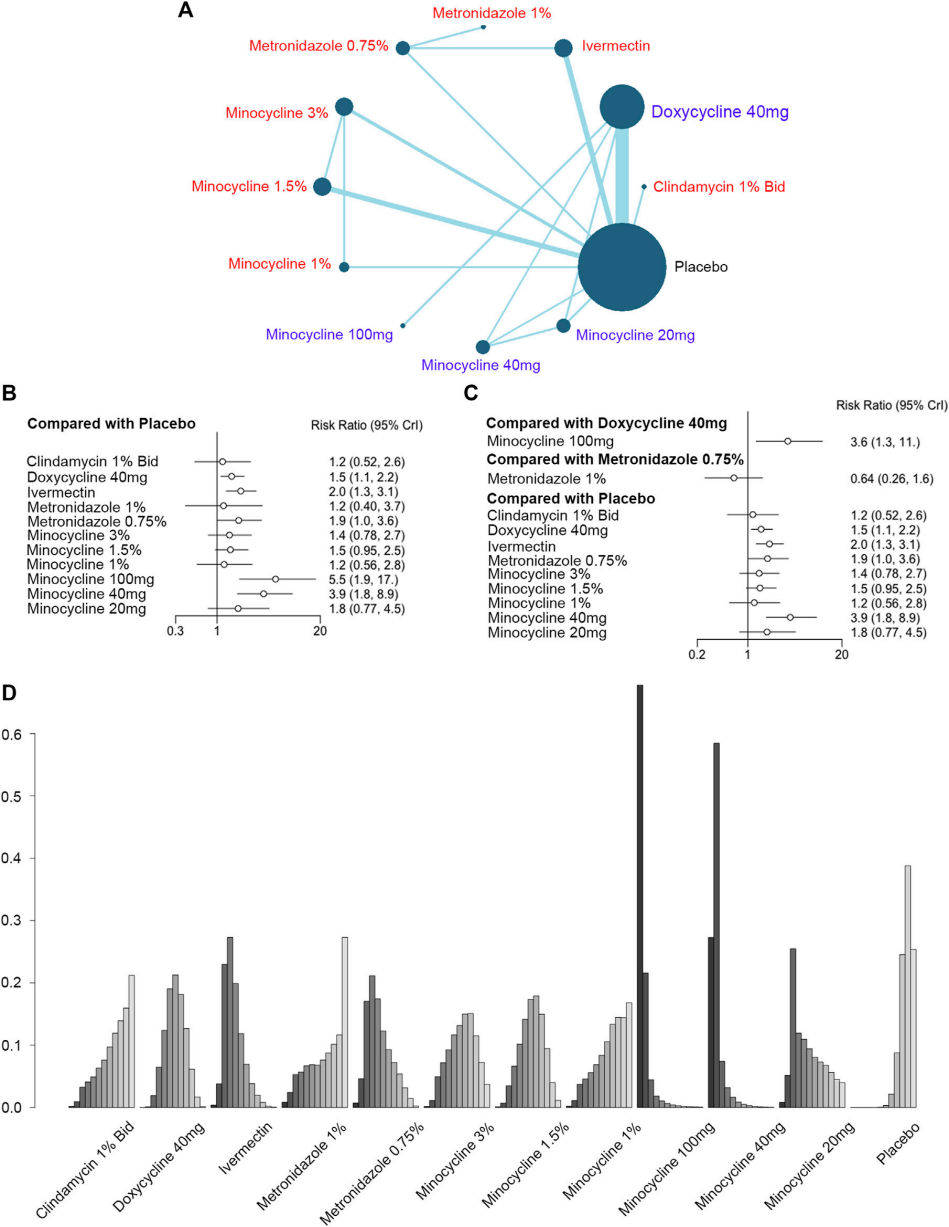
Improvement of the Investigator’s Global Assessment (IGA). **(A)** Network plot of systemic (blue) and topical (red) antibiotic agents improving IGA scores included in the analyses. Each node indicates an intervention, and each direct comparison is represented as lines connecting two nodes. The size of the nodes is proportional to the number of randomly assigned participants. The width of the lines is proportional to the number of randomized clinical trials (RCTs) directly comparing the two connected treatments. Bid = twice a day. **(B)** Forest plot of indirect comparison of antibiotic agents improving IGA scores. **(C)** Forest plot of direct comparison of antibiotic agents improving IGA scores. **(D)** Rank plot of antibiotic agents improving IGA scores.

As for the primary outcome of antibiotics treatment in rosacea, 19 RCTs (6,570 patients) were included in the analysis of IGA improvement, concerning 12 different interventions (Figure 2A). The forest plots showed the indirect and direct comparisons of these treatments. According to Figure 2B and Figure 2C, doxycycline 40 mg [RR, 1.5 (CI, 1.1 to 2.2)], ivermectin [RR, 2.0 (CI, 1.3 to 3.1)], metronidazole 0.75% [RR, 1.9 (CI, 1.0 to 3.6)], minocycline 100 mg [RR, 5.5 (CI, 1.9 to 17)], and minocycline 40 mg [RR, 3.9 (CI, 1.8 to 8.9)] were considered effective when compared to the placebo in improving the IGA scores, and minocycline 100 mg was superior to doxycycline 40 mg [RR, 3.6 (CI, 1.3 to 11)]. There was no difference between metronidazole 0.75% and metronidazole 1% [RR, 0.64 (CI, 0.26 to 1.6)]. The direct and indirect RRs showed no incoherence for the comparison of metronidazole 0.75% *vs.* ivermectin, ivermectin *vs.* placebo, metronidazole 0.75% *vs.* placebo, and minocycline 3% *vs.* minocycline 1.5% (Supplementary Figure S2A). In addition, minocycline 100 mg was ranked as the most effective intervention for reducing the IGA scores, followed by minocycline 40 mg and minocycline 20 mg (Figure 2D; Supplementary Table S5).

### 3.3 Secondary outcomes

#### 3.3.1 Improvement of Patient’s Global Assessment

**FIGURE 3:**
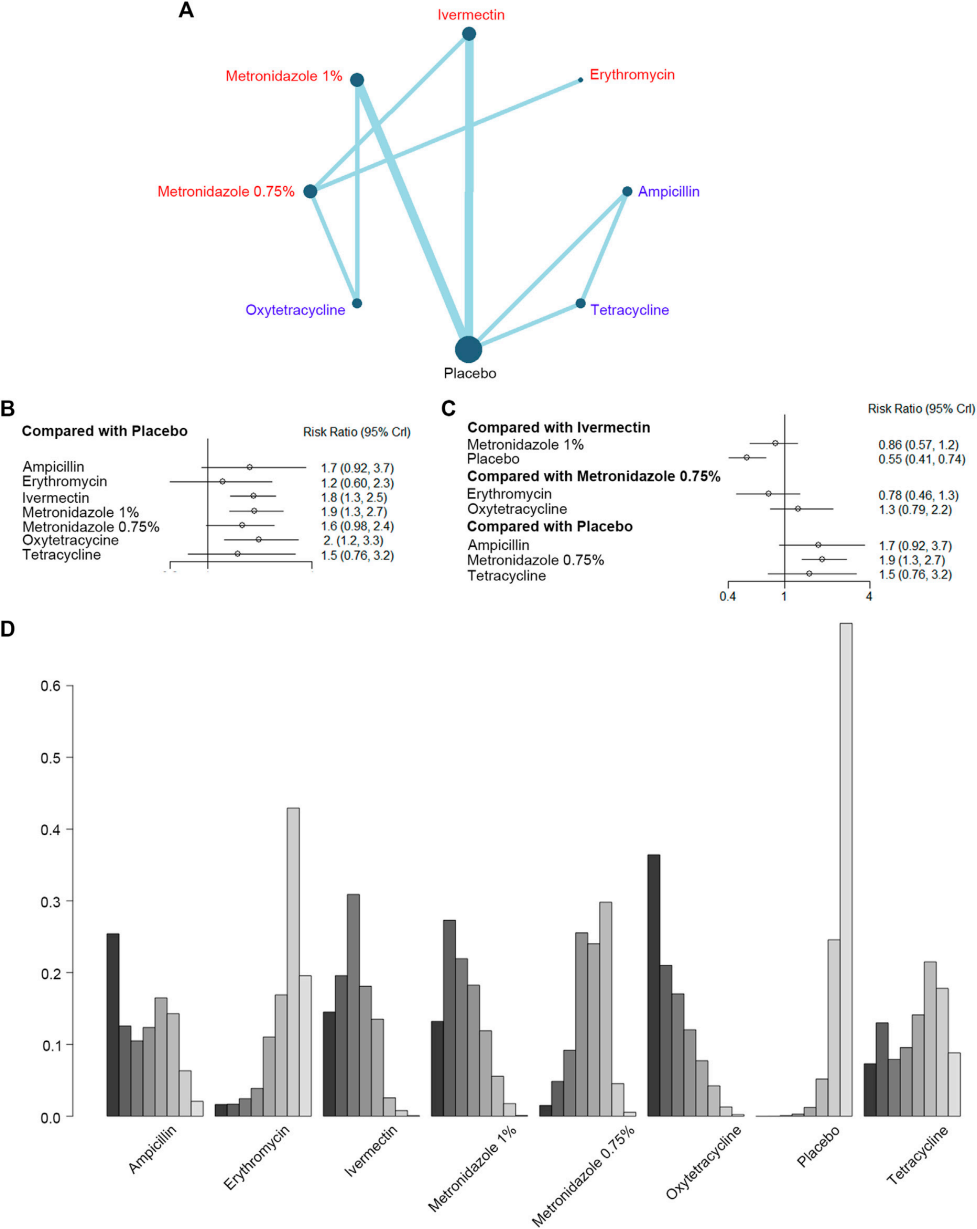
Improvement of the Patient's Global Assessment (PGA). **(A)** Network plot of systemic (blue) and topical (red) antibiotic agents improving PaGA scores included in the analyses. **(B)** Forest plot of indirect comparison of antibiotic agents improving PaGA scores. **(C)** Forest plot of direct comparison of antibiotic agents improving PaGA scores. **(D)** Rank plot of antibiotic agents improving PaGA scores.

Then, we evaluated the effect of eight antibiotics in improving the PaGA scores in nine studies with 2,667 participants (Figure 3A). The forest plots (Figures 3B, C) showed ivermectin [RR, 1.8 (CI, 1.3 to 2.5)], metronidazole 1% [RR, 1.9 (CI, 1.3 to 2.7)], and oxytetracycline [RR, 2 (CI, 1.2 to 3.3)] had significant efficiency when compared to the placebo. Metronidazole 0.75% [RR, 0.86 (CI, 0.57 to 1.2)] was not superior to ivermectin, while erythromycin [RR, 0.78 (CI, 0.46 to 1.3)] and oxytetracycline [RR, 1.3 (CI, 0.79 to 2.2)] did not have significant differences when compared with metronidazole 0.75%. Furthermore, there was no incoherence between the direct and indirect RRs for the comparison of metronidazole 0.75% *vs.* ivermectin, ivermectin *vs.* placebo, oxytetracycline *vs.* metronidazole 1%, metronidazole 1% *vs.* placebo, and oxytetracycline *vs.* metronidazole 0.75% (Supplementary Figure S2B). As for rank probability, oxytetracycline was the best, and to a lesser extent, metronidazole 1% and ivermectin (Figure 3D; Supplementary Table S6).

#### 3.3.2 Reduction of CEA scores

**FIGURE 4:**
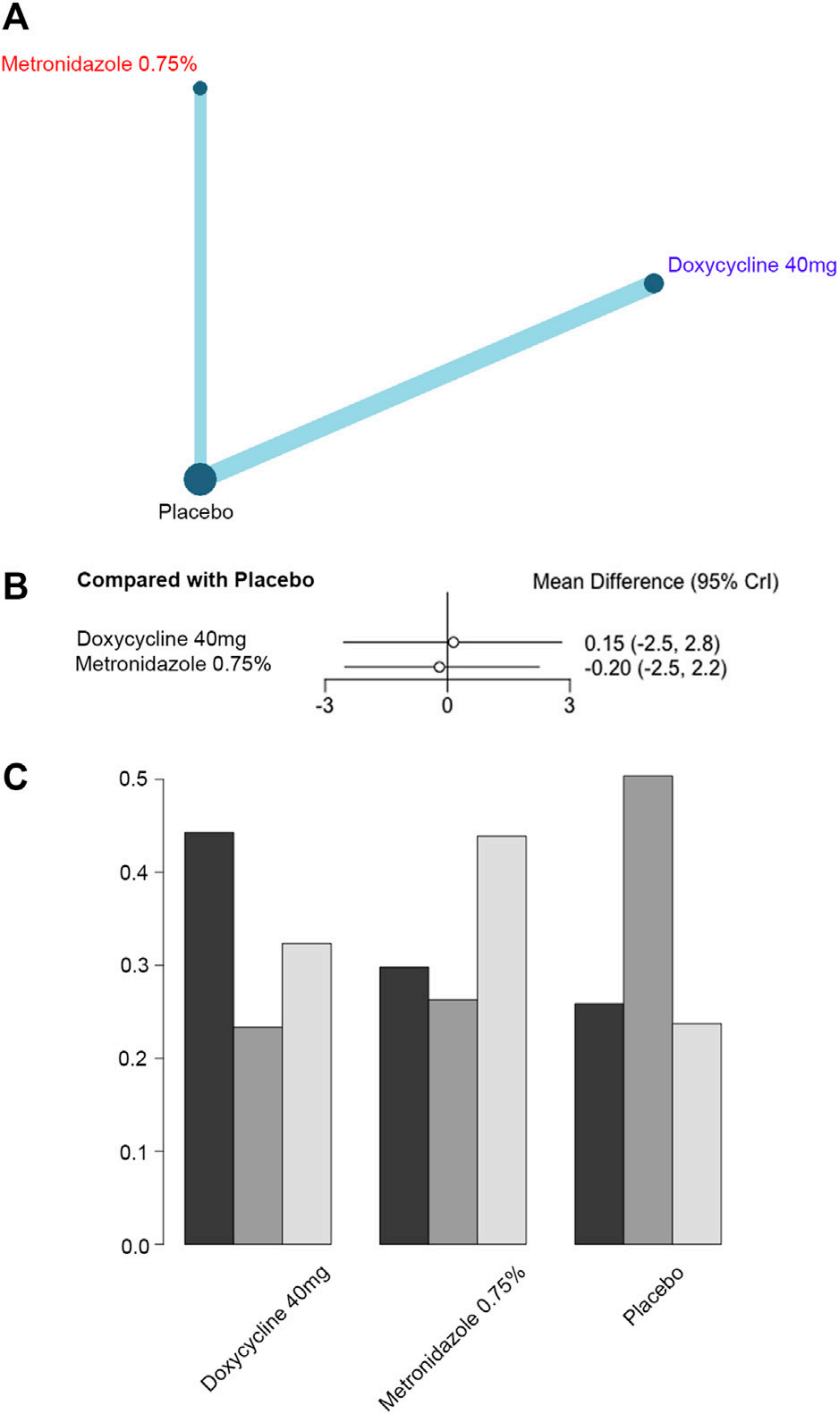
Reduction of Clinician's Erythema Assessment (CEA) scores. **(A)** Network plot of systemic (blue) and topical (red) antibiotic agents improving CEA scores included in the analyses. **(B)** Forest plot of direct comparison of antibiotic agents improving CEA scores. **(C)** Rank plot of antibiotic agents improving CEA scores.

As for the reduction of CEA scores in five trials (743 patients and 3 interventions) (Figures 4A–C), doxycycline 40 mg [MD, 0.15 (CI, −2.5 to 2.8)] and metronidazole 0.75% [MD, −0.20 (CI, −2.5 to 2.2)] were not significantly different from the placebo in decreasing the CEA score.

#### 3.3.3 Report of adverse events

**FIGURE 5:**
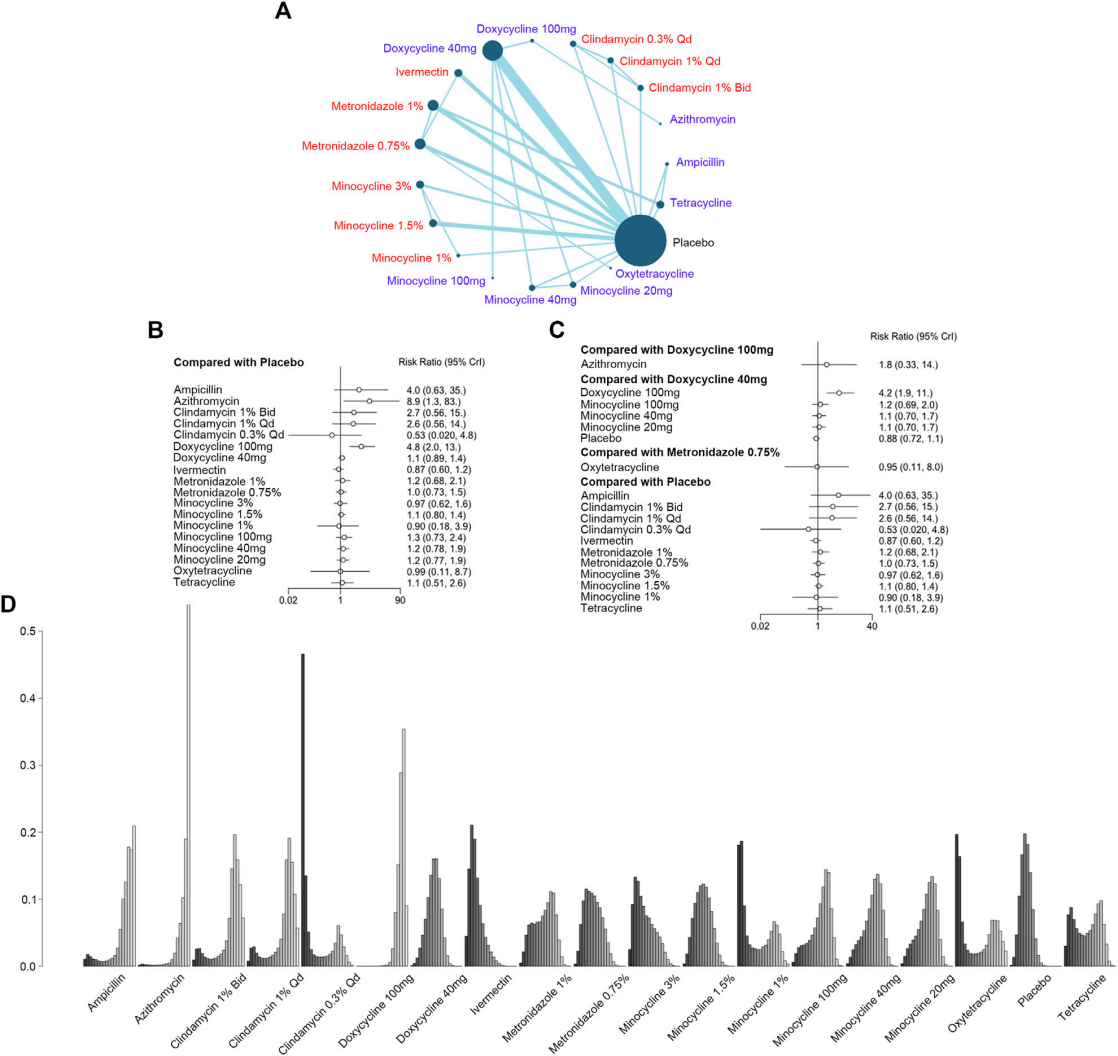
Report of adverse events (AEs). **(A)** Network plot of systemic (blue) and topical (red) antibiotic agents reporting adverse events included in the analyses. Bid = twice a day; qd = once a day. **(B)** Forest plot of indirect comparison of antibiotic agents reporting AEs. **(C)** Forest plot of direct comparison of antibiotic agents reporting AEs. **(D)** Rank plot of antibiotic agents reporting AEs.

Participants with adverse events like epigastric burning were recruited in 30 studies with 19 interventions and 7,872 patients (Figure 5A). As shown in Figure 5, azithromycin [RR, 8.9 (CI, 1.3 to 83)] and doxycycline 100 mg [RR, 4.8 (CI, 2.0 to 13)] increased the risk of AEs when compared with the placebo, while doxycycline 100 mg was associated with a higher risk of AEs when compared with doxycycline 40 mg [RR, 4.2 (CI, 1.9 to 11)] (Figure 5B). However, there was no significant difference between doxycycline 100 mg and azithromycin [RR, 1.8 (CI, 0.33 to 14)] or metronidazole 0.75% and oxytetracycline [RR, 9.5 (CI, 0.11 to 8.0)]. When comparing minocycline 100 mg [RR, 1.2 (CI, 0.69 to 2.0)], minocycline 40 mg [RR, 1.1 (CI, 0.70 to 1.7)], and minocycline 20 mg [RR, 1.1 (CI, 0.70 to 1.7)] with doxycycline 40 mg, the forest plot showed no significant difference in safety (Figure 5C). There was no incoherence between the direct and indirect RRs for the comparison of metronidazole 0.75% *vs.* ivermectin, placebo *vs.* ivermectin, metronidazole 0.75% *vs.* metronidazole 1%, placebo *vs.* metronidazole 1%, tetracycline *vs.* metronidazole 1%, placebo *vs.* metronidazole 0.75%, minocycline 3% *vs.* minocycline 1.5%, and tetracycline *vs.* placebo (Supplementary Figure S2C). Azithromycin was ranked as the most dangerous, with the highest incidence of adverse events, and doxycycline 100 mg ranked the second (Figure 5D; Supplementary Table S7).

Obviously, the topical agents presented various skin irritant reactions. For instance, metronidazole 1% was associated with pruritus, while metronidazole 0.75%, though of lower density, was found to be associated with drying, flaking, stinging, burning, itching, and moderate facial contact dermatitis. Interestingly, metronidazole 1% was also found to correlate with gastrointestinal symptoms (Gamborg Nielsen, 1983; Bitar et al., 1990; Dahl et al., 2001). The treatment-related AEs of 1% minocycline mainly comprised the application site–limited contact dermatitis and pruritus, while that of 1.5% minocycline included pruritus and spotted redness and that of 3% minocycline included eczema, skin exfoliation, pruritus, urticaria, and face burning or stinging (Mrowietz et al., 2018; Gold et al., 2020; Webster et al., 2020). Moreover, topical ivermectin and clindamycin 1% were reported to cause skin irritation and contact dermatitis (Taieb et al., 2015; Martel et al., 2017).

Oral oxytetracycline, tetracycline, azithromycin, and ampicillin were discovered to be related with mild gastrointestinal effects that included diarrhea, constipation, and epigastric burning and burping (Marks and Ellis, 1971; Veien et al., 1986; Akhyani et al., 2008). In addition, oxytetracycline might cause other systemic symptoms. Oral doxycycline 40 mg induced nasopharyngitis, sinusitis, hypertension, and influenza-like symptoms (Del Rosso et al., 2007). Patients treated with minocycline 20 mg might have headache, nasopharyngitis, and back pain, while those treated with minocycline 40 mg might experience headache and nasopharyngitis, and those treated with minocycline 100 mg might experience nausea and headache (Nct, 2016; van der Linden et al., 2017). Interestingly, oral tetracycline might cause folliculitis, though it is prescribed aiming at controlling inflammatory skin lesions (Marks and Ellis, 1971).

## 4 Discussion

This network meta-analysis of 31 trials with 8,226 participants compared the efficacy and safety of antibiotic agents in the treatment of rosacea. The primary outcome was the improvement of inflammatory papules/pustules, evidenced as a decrease in the IGA scores. Meanwhile, a reduction in the PaGA scores represents the overall remission of the disease, while reduction in the CEA scores stands for an alleviation of erythema; the incidence of AEs was the second outcome.

According to the global ROSacea Consensus (ROSCO) panel, the treatment of rosacea is decided mainly based on cutaneous features, such as erythema, inflammatory papules/pustules, telangiectasia, and phyma. Oral doxycycline and topical ivermectin were the first-line treatment for inflammatory papules/pustules, and oral doxycycline was recommended as the first choice for clinically inflamed phyma. Antibiotics that include tetracycline and minocycline have been wildly used among rosacea, since they fit with most situations even when the patients are complicated with several phenotypes. However, doctors should carefully consider benefit and safety when making the prescriptions due to adverse events. In addition, for those with several phenotypes, drug combination therapy could be considered, as it demonstrates higher efficiency, shorter duration, longer remission, and less disease burden. Still, more evidence is required to compare the details of efficiency and side effects among different strategies (Schaller et al., 2017; Schaller et al., 2020; Thiboutot et al., 2020).

Our research indicates that second-generation semi-synthetic tetracyclines, specifically, systemic minocycline and subantimicrobial dose doxycycline, have played an excellent role in reducing papules and pustules. The curative effect of minocycline may be dose dependent, since 100 mg was better than 40 mg, while 20 mg and other topical foams presented no effect. Based on these findings, we inferred that these two kinds of tetracycline may ameliorate the phenotype by systemic effects, which include anti-inflammatory and antibacterial effects, and the inhibition of matrix metalloproteinases phospholipase A2, instead of topical effects such as reducing epidermal hydration (Korting and Schöllmann, 2009; S and Powell, 2014). Among these, in rare cases, minocycline was reported to be associated with side effects that included skin hyperpigmentation, drug-induced hypersensitivity syndrome, and lupus (Rallis et al., 2007; van Zuuren, 2017). On the contrary, doxycycline, regarded as the first-line therapy, was generally thought to have the fewest side effects among all tetracyclines (van Zuuren, 2017). However, according to our results, doxycycline 40 mg showed no difference when compared with systemic minocycline in the number of AEs, while doxycycline 100 mg presented a higher percentage of side effects like epigastric burning (Akhyani et al., 2008). In conclusion, we would recommend the systemic use of minocycline (100 mg) to treat inflammatory lesions in the absence of contraindications.

Although antibiotics are recommended chiefly for phenotypes of papules and pustules in rosacea, we are eager to explore their function on erythema, since flushing and erythema are always present accompanying inflammatory phenotypes. Our findings have suggested that topical metronidazole (0.75%) and oral doxycycline (40 mg) showed no effect in reducing erythema scores. It has been reported that topical minocycline foam is superior to vehicles in decreasing CEA in clinical trials. However, we could not include this study in the analysis due to its different evaluation criteria of “treatment success” (Mrowietz et al., 2018). Based on our previous clinical experience, 100 mg doxycycline might benefit the relieving of flushing and erythema. Still, evidence from clinical trials is urgently required to prove the effect of doxycycline in treating erythema. Owing to the lack of research on the interventions for erythema with antibiotics, we could not provide strong evidence to support the use of antibiotics when dealing with erythema. Brimonidine, oxymetazoline hydrochloride, and other light-based therapy remain the first choices (van Zuuren, 2017).

Clinically, more research-based medical evidence is required to make a prescription specifically targeting the phenotype of rosacea. To date, an integrated systemic review on rosacea treatment has included 106 studies comprising 13,631 patients but without a meta-analysis. A previous review (van Zuuren et al., 2015) reported all the interventions of rosacea up to 2019 by a traditional pairwise comparison based only on direct evidence. Because of the inaccuracy and incompleteness of previous research, we have used a comprehensive research strategy to screen all eligible RCTs. For this reason, we are certain that related trials have not been missed. In addition, most of the studies that have been included are of high quality, especially in the binding methods and data reporting. The low heterogeneity and the consistency of the data further confirm the robustness and accuracy of our results.

### 4.1 Study limitations

This study comprehensively analyzes comparisons of the effect and safety between antibiotics in rosacea therapy, which provides strong evidence to guide clinical treatment. However, there are still some limitations in our study. For instance, the clinical trials have different designs and set up different criteria for the outcomes, which limits the selection when contrasting the treatment, and thus influences the abundance and completeness of the data greatly. In addition, in order to compare the effectiveness and safety of single drug usage more strictly, we did not include combination therapy of antibiotics, which is common in clinical practice.

## 5 Conclusion

In conclusion, our results have suggested minocycline 100 mg is the most effective therapy for rosacea with papules and pustules. Conversely, azithromycin and doxycycline 100 mg should be listed as the last options due to the high proportion of their side effects.

## Data Availability

The original contributions presented in the study are included in the article/Supplementary Material. Further inquiries can be directed to the corresponding author.

## References

[B1] Akhyani M. Ehsani A. H. Ghiasi M. Jafari A. K. (2008). Comparison of efficacy of azithromycin vs. doxycycline in the treatment of rosacea: A randomized open clinical trial. Int. J. Dermatol 47 (3), 284–288. 10.1111/j.1365-4632.2008.03445.x 18289334

[B2] Bitar A. Bourgouin J. Doré N. Dubuc R. Giroux J. M. Landry M. (1990). A double-blind randomised study of metronidazole (Flagyl®) 1% cream in the treatment of acne rosacea. Drug Investig. 2, 242–248. 10.1007/bf03259203

[B28] Bjerke J. R. Nyfors A. Austad J. Rajka G. Gjertsen B. T. Haavelsrud O. (1989). Metronidazole (Elyzol) 1% cream v. placebo cream in the treatment of rosacea. Clin. Trials J. 26 (3), 187–194.

[B29] Cai L. Li W. Xu Q. Du J. Chen Z. Wang L. (2002). Metronidazole gel in treatment of moderate to severe rosacea: A randomized, double-blind, placebo-controlled clinical trial. Zhongguo Xinyao yu Linchuang Zazhi 21 (11), 657–660.

[B3] Dahl M. V. Jarratt M. Kaplan D. Tuley M. R. Baker M. D. (2001). Once-daily topical metronidazole cream formulations in the treatment of the papules and pustules of rosacea. J. Am. Acad. Dermatol 45 (5), 723–730. 10.1067/mjd.2001.116219 11606923

[B4] Del Rosso J. Q. Webster G. F. Jackson M. Rendon M. Rich P. Torok H. (2007). Two randomized phase III clinical trials evaluating anti-inflammatory dose doxycycline (40-mg doxycycline, USP capsules) administered once daily for treatment of rosacea. J. Am. Acad. Dermatol. 56 (5), 791–802. 10.1016/j.jaad.2006.11.021 17367893

[B5] Deng Y. Peng Q. Yang S. Jian D. Wang B. Huang Y. (2018). The Rosacea-specific Quality-of-Life instrument (RosQol): Revision and validation among Chinese patients. PLoS One 13 (2), e0192487. 10.1371/journal.pone.0192487 29489857 PMC5831031

[B30] Fowler J J. F. (2007). Anti-inflammatory dose doxycycline for the treatment of rosacea. Expert Rev. Dermatology 2 (5), 523–531. 10.1586/17469872.2.5.523

[B6] Gamborg Nielsen P. (1983). Treatment of rosacea with i% metronidazole cream. A double-blind study. Br. J. Dermatology 108 (3), 327–332. 10.1111/j.1365-2133.1983.tb03972.x 6219689

[B7] Gether L. Overgaard L. K. Egeberg A. Thyssen J. P. (2018). Incidence and prevalence of rosacea: A systematic review and meta-analysis. Br. J. Dermatol 179 (2), 282–289. 10.1111/bjd.16481 29478264

[B8] Gold L. S. Del Rosso J. Q. Kircik L. Bhatia N. D. Hooper D. Nahm W. K. (2020). Minocycline 1.5% foam for the topical treatment of moderate to severe papulopustular rosacea: Results of 2 phase 3, randomized, clinical trials. J. Am. Acad. Dermatol 82 (5), 1166–1173. 10.1016/j.jaad.2020.01.043 32004648

[B31] Huang E. Y. Di Nardo A. Preston N. J. Gallo R. L. Gottschalk R. W. (2014). Multicenter, randomized, double-blind, placebo-controlled evaluation of rosacea related inflammatory biomarkers in papulopustular rosacea adults treated with doxycycline 40 mg modified release. J. Am. Acad. Dermatology 70 (5), AB9. 10.1016/j.jaad.2014.01.036

[B9] Jacob J. S. Cohen P. R. (2020). Doxycycline-associated dual cutaneous adverse reaction to the drug (CARD): Case report of concurrent photosensitivity and morbilliform exanthem to doxycycline. Cureus 12 (11), e11546. 10.7759/cureus.11546 33365215 PMC7748559

[B32] Koçak M. Yağli S. Vahapoğlu G. Eks¸ioğlu M. (2002). Permethrin 5% cream versus metronidazole 0.75% gel for the treatment of papulopustular rosacea. Dermatol. (basel, Switz. 205 (3), 265–270. 10.1159/000065849 12399675

[B10] Korting H. C. Schöllmann C. (2009). Tetracycline actions relevant to rosacea treatment. Skin. Pharmacol. Physiol. 22 (6), 287–294. 10.1159/000235550 19786821

[B11] Marks R. Ellis J. (1971). Comparative effectiveness of tetracycline and ampicillin in rosacea. A controlled trial. Lancet 2 (7733), 1049–1052. 10.1016/s0140-6736(71)90376-x 4106909

[B12] Martel P. Jarratt M. Weiss J. Carlavan I. (2017). Lack of significant anti-inflammatory activity with clindamycin in the treatment of rosacea: Results of 2 randomized, vehicle-controlled trials. Cutis 100 (1), 53–58. 28873109

[B33] Miyachi Y. Yamasaki K. Fujita T. Fujii C. (2022). Metronidazole gel (0.75%) in Japanese patients with rosacea: A randomized, vehicle-controlled, phase 3 study. J. Dermatology 49 (3), 330–340. 10.1111/1346-8138.16254 PMC9299697 34854112

[B34] Monk B. Logan R. Cook J. White J. Mason R. (1991). Topical metronidazole in the treatment of rosacea. J. Dermatological Treat. 2 (3), 91–93. 10.3109/09546639109092728

[B13] Mrowietz U. Kedem T. H. Keynan R. Eini M. Tamarkin D. Rom D. (2018). A phase II, randomized, double-blind clinical study evaluating the safety, tolerability, and efficacy of a topical minocycline foam, FMX103, for the treatment of facial papulopustular rosacea. Am. J. Clin. Dermatol 19 (3), 427–436. 10.1007/s40257-017-0339-0 29396702

[B14] Nct (2016). Clinical endpoint study of ivermectin 1% cream. Available at; https:// clinicaltrials.gov /show/ nct02840461.

[B35] Nct (2017). A controlled study to assess the efficacy, safety and tolerability of oral DFD-29 extended release capsules. Available at: http://cochranelibrary-wiley.com/o/cochrane/clcentral/articles/764/CN-01565764/frame.html.

[B15] Rallis E. Korfitis C. Gregoriou S. Rigopoulos D. (2007). Assigning new roles to topical tacrolimus. Expert Opin. Investigational Drugs 16 (8), 1267–1276. 10.1517/13543784.16.8.1267 17685874

[B16] S N. R. Powell F. C. (2014). Epidermal hydration levels in patients with rosacea improve after minocycline therapy. Br. J. Dermatol 171 (2), 259–266. 10.1111/bjd.12770 24354646

[B36] Schachter D. Schachter R. Long B. Shiffman N. Lester R. Miller S. (1991). Comparison of metronidazole 1% cream versus oral tetracycline in patients with rosacea. Drug Investig. 3 (4), 220–224. 10.1007/bf03259568

[B17] Schaller M. Almeida L. M. C. Bewley A. Cribier B. Del Rosso J. Dlova N. C. (2020). Recommendations for rosacea diagnosis, classification and management: Update from the global ROSacea COnsensus 2019 panel. Br. J. Dermatol 182 (5), 1269–1276. 10.1111/bjd.18420 31392722 PMC7317217

[B18] Schaller M. Almeida L. M. C. Bewley A. Cribier B. Dlova N. C. Kautz G. (2017). Rosacea treatment update: Recommendations from the global ROSacea COnsensus (ROSCO) panel. Br. J. Dermatol 176 (2), 465–471. 10.1111/bjd.15173 27861741

[B19] Smilack J. D. (1999). The tetracyclines. Mayo Clin. Proc. 74 (7), 727–729. 10.4065/74.7.727 10405705

[B37] Stein L. Kircik L. Fowler J. Tan J. Draelos Z. Fleischer A. (2014). Efficacy and safety of ivermectin 1% cream in treatment of papulopustular rosacea: Results of two randomized, double-blind, vehicle-controlled pivotal studies. J. Drugs Dermatology 13 (3), 316–323. 10.3410/f.718301436.793495893 24595578

[B20] Taieb A. Ortonne J. P. Ruzicka T. Roszkiewicz J. Berth-Jones J. Peirone M. H. (2015). Superiority of ivermectin 1% cream over metronidazole 0·75% cream in treating inflammatory lesions of rosacea: A randomized, investigator-blinded trial. Br. J. Dermatol 172 (4), 1103–1110. 10.1111/bjd.13408 25228137

[B21] Tan J. Berg M. Gallo R. L. Del Rosso J. Q. (2018). Applying the phenotype approach for rosacea to practice and research. Br. J. Dermatol 179 (3), 741–746. 10.1111/bjd.16815 29799114

[B22] Thiboutot D. Anderson R. Cook-Bolden F. Draelos Z. Gallo R. L. Granstein R. D. (2020). Standard management options for rosacea: The 2019 update by the national rosacea society Expert committee. J. Am. Acad. Dermatol 82 (6), 1501–1510. 10.1016/j.jaad.2020.01.077 32035944

[B23] Van der Linden M. M. D. Van Ratingen A. R. Van Rappard D. C. Nieuwenburg S. A. Spuls P. I. (2017). DOMINO, doxycycline 40 mg vs. minocycline 100 mg in the treatment of rosacea: A randomized, single-blinded, noninferiority trial, comparing efficacy and safety. Br. J. Dermatol 176 (6), 1465–1474. 10.1111/bjd.15155 27797396

[B24] Van Zuuren E. J. Graber M. A. Hollis S. Chaudhry M. Gupta A. K. Gover M. (2015). Interventions for rosacea. Cochrane Database Syst. Rev. 2015 (4), Cd003262. 10.1002/14651858.CD003262.pub3 16034895

[B25] Van Zuuren E. J. (2017). Rosacea. N. Engl. J. Med. 377 (18), 1754–1764. 10.1056/NEJMcp1506630 29091565

[B26] Veien N. K. Christiansen J. V. Hjorth N. Schmidt H. (1986). Topical metronidazole in the treatment of rosacea. Cutis 38 (3), 209–210. 2945705

[B38] Verea H. M. Margusino F. L. Seco V. C. Feal C. B. Cuna E. B. (1992). Comparative study of topical erythromycin and topical metronidazole in the treatment of rosacea. Farm. Clin. 9 (6), 472–479.

[B27] Webster G. Draelos Z. D. Graber E. Lee M. S. Dhawan S. Salman M. (2020). A multicentre, randomized, double-masked, parallel group, vehicle-controlled phase IIb study to evaluate the safety and efficacy of 1% and 3% topical minocycline gel in patients with papulopustular rosacea. Br. J. Dermatol 183 (3), 471–479. 10.1111/bjd.18857 31907924 PMC7496252

