# Supplementary material for: Efficacy and safety of antibiotic agents in the treatment of rosacea: a systemic network meta-analysis
Source: Front Pharmacol. 2023 May 11;14:1169916. doi: 10.3389/fphar.2023.1169916 (PMC10210163; doi:10.3389/fphar.2023.1169916)
Supplement: Supplementary file 1 [file Table1.docx]

Supplementary Material

Efficacy and safety of antibiotic agents in the treatment of Rosacea: a systemic network meta-analysis

Wenqin Xiao, Mengting Chen, Ben Wang, Yingxue Huang, Zhixiang Zhao, Zhili Deng, Hongfu Xie, Ji Li, Yan Tang^*^

*** Correspondence:** Yan Tang: [ytang_xy@csu.edu.cn](mailto:ytang_xy@csu.edu.cn)

# Tables

## Table S1. Analysis of heterogeneity in IGA score

Per-comparison I-squared:

-------------------------

| t1 | t2 | i2.pair | i2.cons | incons.p |
| --- | --- | --- | --- | --- |
| Clindamycin_a | Placebo | NA | NA | NA |
| Doxycycline_b | Minocycline_d | NA | NA | NA |
| Doxycycline_b | Minocycline_e | NA | NA | NA |
| Doxycycline_b | Minocycline_f | NA | NA | NA |
| Doxycycline_b | Placebo | 0.00000 | 0.00000 | NA |
| Ivermectin | Metronidazole_b | NA | 49.44427 | 0.4820781 |
| Ivermectin | Placebo | 90.06466 | 85.84421 | 0.4661570 |
| Metronidazole_a | Metronidazole_b | NA | NA | NA |
| Metronidazole_b | Placebo | NA | 30.47853 | 0.3561204 |
| Minocycline_a | Minocycline_b | NA | 30.07507 | 0.3364990 |
| Minocycline_a | Minocycline_c | NA | NA | NA |
| Minocycline_a | Placebo | 0.00000 | 0.00000 | NA |
| Minocycline_b | Placebo | 46.39584 | 61.26989 | NA |
| Minocycline_c | Placebo | NA | NA | NA |
| Minocycline_e | Minocycline_f | NA | NA | NA |
| Minocycline_e | Placebo | NA | NA | NA |
| Minocycline_f | Placebo | NA | NA | NA |

Global I-squared:

-------------------------

| i2.pair | i2.cons |
| --- | --- |
| 60.47707 | 51.76024 |

## Table S2. Analysis of heterogeneity in PaGA score

Per-comparison I-squared:

-------------------------

| t1 | t2 | i2.pair | i2.cons | incons.p |
| --- | --- | --- | --- | --- |
| Ampicillin | Placebo | NA | NA | NA |
| Ampicillin | Tetracycline | NA | NA | NA |
| Erythromycin | Metronidazole_b | NA | NA | NA |
| Ivermectin | Metronidazole_b | NA | 0 | 0.9539280 |
| Ivermectin | Placebo | 0 | 0 | 0.9692857 |
| Metronidazole_a | Oxytetracycline | NA | 0 | 0.7163108 |
| Metronidazole_a | Placebo | 0 | 0 | 0.8456203 |
| Metronidazole_b | Oxytetracycline | NA | 0 | 0.7344033 |
| Placebo | Tetracycline | NA | NA | NA |

Global I-squared:

-------------------------

| i2.pair | i2.cons |
| --- | --- |
| 0 | 0 |

## Table S3. Analysis of heterogeneity in CEA score

Per-comparison I-squared:

-------------------------

| t1 | t2 | i2.pair | i2.cons | incons.p |
| --- | --- | --- | --- | --- |
| Doxycycline_b | Placebo | 0 | 0 | NA |
| Metronidazole_b | Placebo | 0 | 0 | NA |

Global I-squared:

-------------------------

| i2.pair | i2.cons |
| --- | --- |
| 0 | 0 |

## Table S4. Analysis of heterogeneity in adverse event

Per-comparison I-squared:

-------------------------

| t1 | t2 | i2.pair | i2.cons | incons.p |
| --- | --- | --- | --- | --- |
| Ampicillin | Placebo | NA | NA | NA |
| Ampicillin | Tetracycline | NA | NA | NA |
| Azithromycin | Doxycycline_a | NA | NA | NA |
| Clindamycin_a | Clindamycin_b | NA | NA | NA |
| Clindamycin_a | Clindamycin_c | NA | NA | NA |
| Clindamycin_a | Placebo | NA | NA | NA |
| Clindamycin_b | Clindamycin_c | NA | NA | NA |
| Clindamycin_b | Placebo | NA | NA | NA |
| Clindamycin_c | Placebo | NA | NA | NA |
| Doxycycline_a | Doxycycline_b | NA | NA | NA |
| Doxycycline_b | Minocycline_d | NA | NA | NA |
| Doxycycline_b | Minocycline_e | NA | NA | NA |
| Doxycycline_b | Minocycline_f | NA | NA | NA |
| Doxycycline_b | Placebo | 14.123364 | 16.26939 | NA |
| Ivermectin | Metronidazole_b | NA | 72.18744 | 0.22187437 |
| Ivermectin | Placebo | 1.713192 | 57.66264 | 0.02907023 |
| Metronidazole_a | Metronidazole_b | NA | 0.00000 | 0.90282812 |
| Metronidazole_a | Placebo | 0.000000 | 0.00000 | 0.83262461 |
| Metronidazole_a | Tetracycline | 32.506853 | 32.37151 | NA |
| Metronidazole_b | Oxytetracycline | NA | NA | NA |
| Metronidazole_b | Placebo | 0.000000 | 0.00000 | 0.14689282 |
| Minocycline_a | Minocycline_b | NA | 76.69055 | 0.05856607 |
| Minocycline_a | Minocycline_c | NA | NA | NA |
| Minocycline_a | Placebo | 13.664518 | 30.69477 | NA |
| Minocycline_b | Placebo | 57.725354 | 66.01118 | NA |
| Minocycline_c | Placebo | NA | NA | NA |
| Minocycline_e | Minocycline_f | NA | NA | NA |
| Minocycline_e | Placebo | NA | NA | NA |
| Minocycline_f | Placebo | NA | NA | NA |
| Placebo | Tetracycline | NA | 0.00000 | 0.94795258 |

Global I-squared:

-------------------------

| i2.pair | i2.cons |
| --- | --- |
| 11.35889 | 0 |

## Table S5. Rank probability in IGA score

|  | 1 | 2 | 3 | 4 | 5 | 6 | 7 | 8 | 9 | 10 | 11 | 12 |
| --- | --- | --- | --- | --- | --- | --- | --- | --- | --- | --- | --- | --- |
| Clindamycin_a | 0.001900 | 0.009455 | 0.032590 | 0.040955 | 0.049125 | 0.063220 | 0.075915 | 0.097095 | 0.119210 | 0.139080 | 0.159470 | 0.211985 |
| Doxycycline_b | 0.000015 | 0.000995 | 0.019295 | 0.064800 | 0.123760 | 0.190475 | 0.212690 | 0.181515 | 0.126640 | 0.061605 | 0.016740 | 0.001470 |
| Ivermectin | 0.003810 | 0.038010 | 0.229615 | 0.272970 | 0.199060 | 0.118585 | 0.069270 | 0.038275 | 0.019810 | 0.008035 | 0.002140 | 0.000420 |
| Metronidazole_a | 0.008445 | 0.024245 | 0.052815 | 0.056885 | 0.066975 | 0.068555 | 0.068030 | 0.076025 | 0.087390 | 0.116475 | 0.101215 | 0.272945 |
| Metronidazole_b | 0.007315 | 0.046080 | 0.170535 | 0.211450 | 0.174360 | 0.122815 | 0.092870 | 0.072160 | 0.053770 | 0.031670 | 0.014665 | 0.002310 |
| Minocycline_a | 0.001620 | 0.011190 | 0.049480 | 0.071795 | 0.092510 | 0.116550 | 0.131410 | 0.149960 | 0.150890 | 0.115085 | 0.072335 | 0.037175 |
| Minocycline_b | 0.000875 | 0.007115 | 0.034905 | 0.066185 | 0.101570 | 0.141410 | 0.173320 | 0.179030 | 0.149765 | 0.094615 | 0.039910 | 0.011300 |
| Minocycline_c | 0.002095 | 0.011125 | 0.037305 | 0.045590 | 0.055915 | 0.068580 | 0.083545 | 0.105575 | 0.133510 | 0.144685 | 0.143950 | 0.168125 |
| Minocycline_d | 0.692985 | 0.215910 | 0.044555 | 0.018240 | 0.010465 | 0.006460 | 0.004070 | 0.002770 | 0.001870 | 0.001360 | 0.000800 | 0.000515 |
| Minocycline_e | 0.272805 | 0.584315 | 0.074145 | 0.031865 | 0.016540 | 0.008745 | 0.005115 | 0.003000 | 0.001775 | 0.000940 | 0.000490 | 0.000265 |
| Minocycline_f | 0.008135 | 0.051560 | 0.254755 | 0.119260 | 0.109650 | 0.094250 | 0.080440 | 0.073080 | 0.067650 | 0.056310 | 0.045090 | 0.039820 |
| Placebo | 0.000000 | 0.000000 | 0.000005 | 0.000005 | 0.000070 | 0.000355 | 0.003325 | 0.021515 | 0.087720 | 0.245400 | 0.387935 | 0.253670 |

## Table S6. Rank probability in PaGA score

|  | 1 | 2 | 3 | 4 | 5 | 6 | 7 | 8 |
| --- | --- | --- | --- | --- | --- | --- | --- | --- |
| Ampicillin | 0.245380 | 0.126030 | 0.110685 | 0.124600 | 0.167065 | 0.138845 | 0.064200 | 0.023195 |
| Erythromycin | 0.016580 | 0.017220 | 0.023730 | 0.038770 | 0.111355 | 0.166835 | 0.419715 | 0.205795 |
| Ivermectin | 0.152770 | 0.191585 | 0.296555 | 0.190295 | 0.132660 | 0.027025 | 0.008475 | 0.000635 |
| Metronidazole_a | 0.135330 | 0.275345 | 0.224390 | 0.179505 | 0.114340 | 0.053750 | 0.016430 | 0.000910 |
| Metronidazole_b | 0.015070 | 0.050485 | 0.090830 | 0.251685 | 0.246620 | 0.292980 | 0.046500 | 0.005830 |
| Oxytetracycline | 0.365985 | 0.214110 | 0.174880 | 0.116215 | 0.073910 | 0.039125 | 0.013385 | 0.002390 |
| Placebo | 0.000000 | 0.000100 | 0.000715 | 0.003095 | 0.012055 | 0.055570 | 0.256030 | 0.672435 |
| Tetracycline | 0.068885 | 0.125125 | 0.078215 | 0.095835 | 0.141995 | 0.225870 | 0.175265 | 0.088810 |

## Table S7. Rank probability in adverse event

|  |  |  |  |  |  |  |  |  |  |  |  |  |  |  |  |  |  |  |  |
| --- | --- | --- | --- | --- | --- | --- | --- | --- | --- | --- | --- | --- | --- | --- | --- | --- | --- | --- | --- |
| Ampicillin | 0.010490 | 0.017700 | 0.014225 | 0.010780 | 0.010090 | 0.007990 | 0.007145 | 0.007230 | 0.007860 | 0.009775 | 0.011885 | 0.015850 | 0.027200 | 0.055180 | 0.100365 | 0.125590 | 0.177710 | 0.173495 | 0.209440 |
| Azithromycin | 0.001670 | 0.003090 | 0.002430 | 0.002295 | 0.001730 | 0.001535 | 0.001720 | 0.001880 | 0.002155 | 0.002735 | 0.003645 | 0.004950 | 0.009985 | 0.019525 | 0.042160 | 0.063750 | 0.101850 | 0.189860 | 0.543035 |
| Clindamycin_a | 0.009430 | 0.025795 | 0.026370 | 0.018960 | 0.013815 | 0.011625 | 0.010275 | 0.010780 | 0.012305 | 0.014965 | 0.018025 | 0.023905 | 0.037990 | 0.071250 | 0.145300 | 0.196555 | 0.158930 | 0.121700 | 0.072025 |
| Clindamycin_b | 0.007690 | 0.027100 | 0.028830 | 0.019715 | 0.013730 | 0.011965 | 0.011820 | 0.012275 | 0.013745 | 0.016485 | 0.020765 | 0.027430 | 0.040530 | 0.078115 | 0.158810 | 0.191245 | 0.155190 | 0.107530 | 0.057030 |
| Clindamycin_c | 0.465820 | 0.134900 | 0.051070 | 0.025120 | 0.017180 | 0.014660 | 0.013475 | 0.013640 | 0.014120 | 0.015205 | 0.017985 | 0.022660 | 0.034575 | 0.060615 | 0.046880 | 0.028750 | 0.015830 | 0.005645 | 0.001870 |
| Doxycycline_a | 0.000010 | 0.000035 | 0.000040 | 0.000120 | 0.000070 | 0.000085 | 0.000140 | 0.000210 | 0.000275 | 0.000475 | 0.000850 | 0.001780 | 0.005655 | 0.025960 | 0.080310 | 0.151660 | 0.288370 | 0.353690 | 0.090265 |
| Doxycycline_b | 0.000700 | 0.004515 | 0.012500 | 0.027180 | 0.046445 | 0.070455 | 0.102665 | 0.135970 | 0.160200 | 0.160265 | 0.130800 | 0.085405 | 0.042530 | 0.015560 | 0.004030 | 0.000715 | 0.000060 | 0.000005 | 0.000000 |
| Ivermectin | 0.044695 | 0.145155 | 0.210390 | 0.189705 | 0.131670 | 0.090910 | 0.063495 | 0.043010 | 0.031130 | 0.021315 | 0.013870 | 0.008040 | 0.004195 | 0.001745 | 0.000515 | 0.000125 | 0.000030 | 0.000005 | 0.000000 |
| Metronidazole_a | 0.004995 | 0.021455 | 0.046440 | 0.061365 | 0.064155 | 0.062875 | 0.065690 | 0.066235 | 0.075145 | 0.083515 | 0.094775 | 0.111315 | 0.109090 | 0.076505 | 0.039265 | 0.014390 | 0.002615 | 0.000170 | 0.000005 |
| Metronidazole_b | 0.003130 | 0.022460 | 0.062315 | 0.097550 | 0.115295 | 0.111775 | 0.109060 | 0.104960 | 0.095515 | 0.087310 | 0.072495 | 0.055425 | 0.035230 | 0.018470 | 0.006850 | 0.001850 | 0.000265 | 0.000045 | 0.000000 |
| Minocycline_a | 0.025210 | 0.091980 | 0.132965 | 0.126810 | 0.104280 | 0.089490 | 0.075465 | 0.071985 | 0.062655 | 0.056000 | 0.050530 | 0.043495 | 0.033810 | 0.020590 | 0.010215 | 0.003650 | 0.000755 | 0.000090 | 0.000025 |
| Minocycline_b | 0.003445 | 0.018235 | 0.043205 | 0.071235 | 0.093975 | 0.109995 | 0.120075 | 0.122760 | 0.118285 | 0.101640 | 0.081520 | 0.056130 | 0.034690 | 0.016680 | 0.006180 | 0.001655 | 0.000270 | 0.000020 | 0.000005 |
| Minocycline_c | 0.180695 | 0.186620 | 0.090165 | 0.045405 | 0.032180 | 0.027575 | 0.026020 | 0.024830 | 0.025165 | 0.028895 | 0.031440 | 0.039810 | 0.052440 | 0.066470 | 0.060930 | 0.047985 | 0.022100 | 0.008000 | 0.003275 |
| Minocycline_d | 0.005875 | 0.018885 | 0.027990 | 0.030690 | 0.031765 | 0.034080 | 0.038375 | 0.046345 | 0.057345 | 0.072295 | 0.093445 | 0.118330 | 0.144110 | 0.139695 | 0.086035 | 0.042760 | 0.010585 | 0.001220 | 0.000175 |
| Minocycline_e | 0.003880 | 0.013410 | 0.024595 | 0.032360 | 0.038315 | 0.043750 | 0.053115 | 0.067210 | 0.085845 | 0.106180 | 0.129845 | 0.137010 | 0.123070 | 0.083515 | 0.041095 | 0.013865 | 0.002570 | 0.000345 | 0.000025 |
| Minocycline_f | 0.003875 | 0.014945 | 0.026105 | 0.033085 | 0.039140 | 0.045145 | 0.054895 | 0.067355 | 0.085095 | 0.107605 | 0.125075 | 0.133975 | 0.122955 | 0.083305 | 0.040120 | 0.014415 | 0.002685 | 0.000195 | 0.000030 |
| Oxytetracycline | 0.196820 | 0.163830 | 0.065975 | 0.033245 | 0.023570 | 0.019195 | 0.018080 | 0.018360 | 0.019505 | 0.021090 | 0.024890 | 0.031175 | 0.047355 | 0.068665 | 0.068255 | 0.068075 | 0.052615 | 0.036665 | 0.022635 |
| Placebo | 0.001250 | 0.013270 | 0.046695 | 0.104685 | 0.166560 | 0.197580 | 0.181990 | 0.139495 | 0.084605 | 0.040930 | 0.016115 | 0.005185 | 0.001295 | 0.000280 | 0.000065 | 0.000000 | 0.000000 | 0.000000 | 0.000000 |
| Tetracycline | 0.030320 | 0.076620 | 0.087695 | 0.069695 | 0.056035 | 0.049315 | 0.046500 | 0.045470 | 0.049050 | 0.053320 | 0.062045 | 0.078130 | 0.093295 | 0.097875 | 0.062620 | 0.032965 | 0.007570 | 0.001320 | 0.000160 |

# Figures


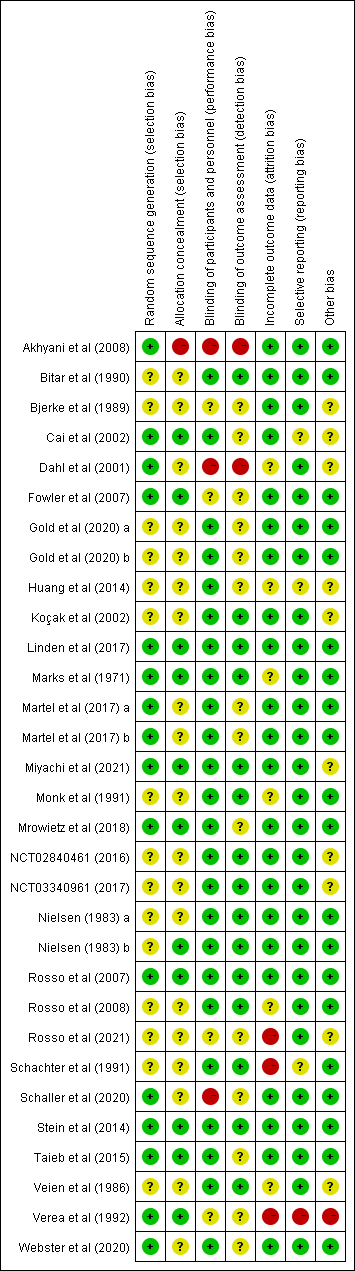


## Figure. S1 The Risk of bias of studies included


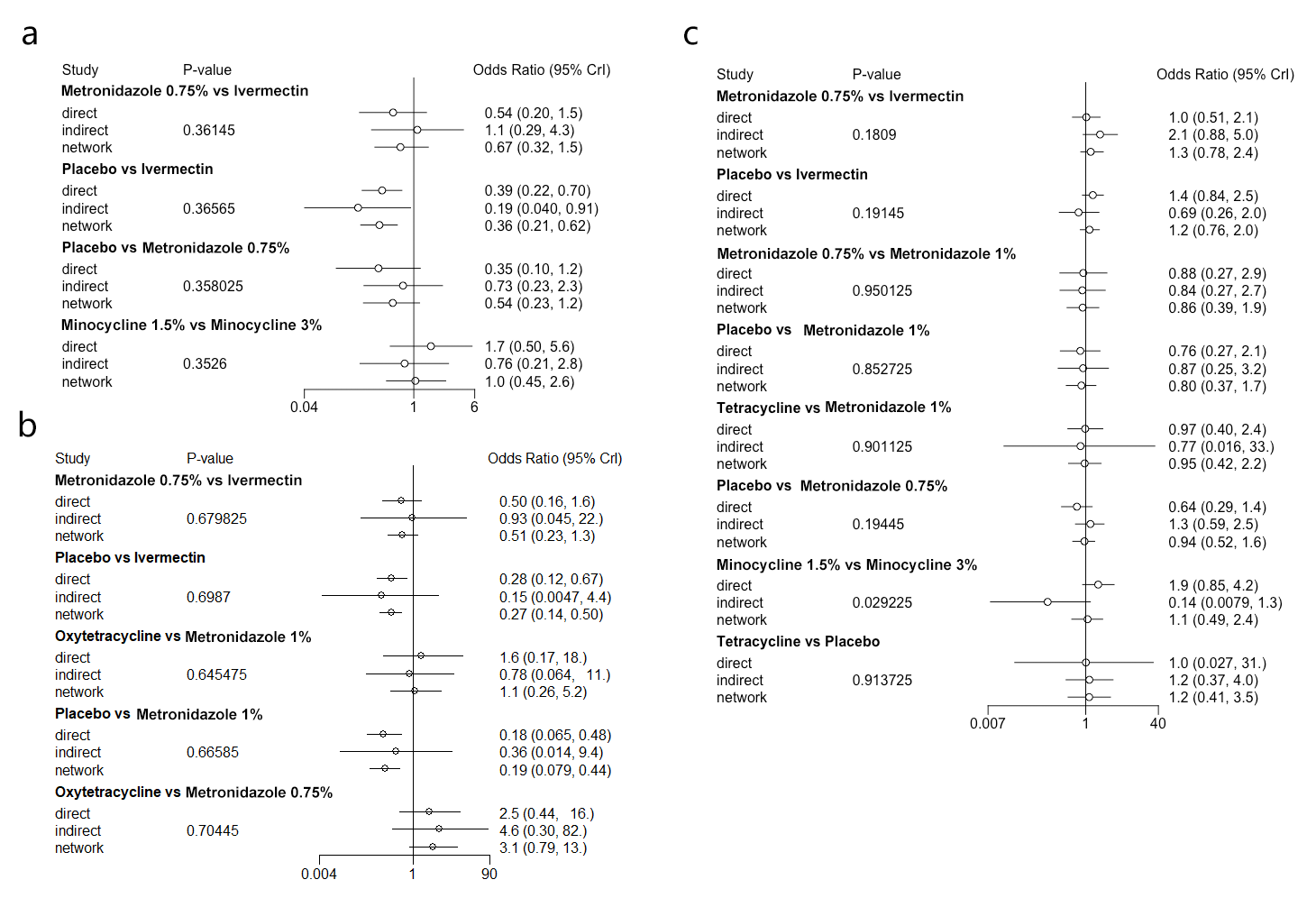


## Figure. S2 The Node-splitting analysis of inconsistency of antibiotic agents included. a. Antibiotics involved in Investigator’s Global Assessment; b. Antibiotics involved in Patient’s Global Assessment; c. Antibiotics involved in Adverse Events.
